# Embedded Temporal Convolutional Networks for Essential Climate Variables Forecasting

**DOI:** 10.3390/s22051851

**Published:** 2022-02-26

**Authors:** Maria Myrto Villia, Grigorios Tsagkatakis, Mahta Moghaddam, Panagiotis Tsakalides

**Affiliations:** 1 Institute of Computer Science, Foundation for Research and Technology-Hellas (FORTH), 70013 Heraklion, Greece; villiamyrto@gmail.com (M.M.V.); tsakalid@ics.forth.gr (P.T.); 2 Department of Electrical Engineering, University of Southern California, Los Angeles, CA 90089, USA; mahta@usc.edu; 3 Computer Science Department, University of Crete, 70013 Hraklion, Greece

**Keywords:** deep learning, time-series forecasting, remote sensing, climate variables, surface temperature, soil moisture

## Abstract

Forecasting the values of essential climate variables like land surface temperature and soil moisture can play a paramount role in understanding and predicting the impact of climate change. This work concerns the development of a deep learning model for analyzing and predicting spatial time series, considering both satellite derived and model-based data assimilation processes. To that end, we propose the Embedded Temporal Convolutional Network (E-TCN) architecture, which integrates three different networks, namely an encoder network, a temporal convolutional network, and a decoder network. The model accepts as input satellite or assimilation model derived values, such as land surface temperature and soil moisture, with monthly periodicity, going back more than fifteen years. We use our model and compare its results with the state-of-the-art model for spatiotemporal data, the ConvLSTM model. To quantify performance, we explore different cases of spatial resolution, spatial region extension, number of training examples and prediction windows, among others. The proposed approach achieves better performance in terms of prediction accuracy, while using a smaller number of parameters compared to the ConvLSTM model. Although we focus on two specific environmental variables, the method can be readily applied to other variables of interest.

## 1. Introduction

Climate change is one of the biggest challenges of modern civilization, manifested by phenomena like extreme weather events, sea level rise, shrinking of ice sheets, warming of the oceans, and more [1]. These changes have been observed since the middle of the 20th century, however, in the past twenty years, the dramatic increase in the number and capabilities of Earth Observation platforms have facilitated a much deeper understanding of the involved processes [2]. To that end, a list of critical environmental parameters has been identified as Essential Climate Variables (ECV) [3], and include soil moisture, land surface temperature, above-ground biomass, sea-level, etc.

Estimation of these variables typically relies on numerical simulation models running on high-performance computing systems [4]. In recent years, however, the dramatic increase in computational capabilities offered by novel hardware platforms like graphical processing units, have allowed cutting edge machine learning algorithms to be introduced to the analysis of Earth observations, contributing to the prediction, recognition and mitigation of climate change [5]. The success of machine learning methods on the analysis of remote sensing observation has been primarily focused on either the classification of static observations [6,7], the enhancement of low quality observations [8,9] or the retrieval of bio/geophysical parameters [10,11].

While significant effort has been allocated to the analysis of the instantaneous values of ECVs, aspects related to understanding the dynamic nature of the observations mandate novel functionalities, like the ability to perform accurate and timely forecast of critical geophysical parameters. In this work, we propose the Embedded Temporal Convolutional Network (E-TCN), a novel deep learning framework for encoding and forecasting ECV and more specifically surface soil moisture and surface temperature. The main contributions of this work are summarized as follows:

- We propose a novel deep learning architecture for forecasting future values which gracefully handles the high-dimensionality of observations.
- We introduce novel datasets of satellite derived geophysical parameters, namely land surface temperature and surface soil moisture, obtained on monthly periodicity over 17.5 years.
- We performed a detailed analysis of both state-of-the-art and proposed deep learning models for the problem of climate variables prediction.

This paper presents related research in Section 2, the Embedded Temporal Convolutional Network in Section 3.1 and the datasets in Section 3.2. In Section 4, we explain the experimental procedure, we show the results of our model and we perform a comparison with the ConvLSTM model. A discussion is presented in Section 5 and the paper concludes in Section 6.

## 2. Related Work

The analysis of spatiotemporal data, i.e., sequences of spatial observations, is attracting a lot of attention lately in Earth Observation due to the numerous applications which can benefit from such data including water, carbon and biochemical cycles. In the deep learning community, problems with sequential dependence are usually approached with Recurrent Neural Networks (RNNs) and Long Short Term Memory networks (LSTMs) [12] due to their capability to capture temporal dependencies in sequential data. There are many variants of these networks that appear in the literature [13,14] and are applied on a wide range of sequential problems such as time series prediction [15], speech recognition [16] and machine translation [17] among others.

For the case of remote sensing, the majority of effort has been allocated to the introduction of deep learning models for supervised classification problems like scene categorization [7], land cover classification [18], and building extraction [19] amongst many others. For the problem of geophysical parameters value retrieval from current observations, more limited effort has been given to specific cases like soil moisture [20], land surface temperature [21], and chlorophyll-a concentration [10], among others. More recently, the analysis of spatio-temporal observation was considered in [22] for the problem of precipitation nowcasting. The goal in this case is to predict the future radar maps by giving as input a past radar echo sequence. Thus, the input and the target output are both spatiotemporal sequences. To that end, the paper proposes the ConvLSTM model, a LSTM architecture which employs convolutions instead of matrix multiplications in the input-to-state and state-to-state transitions. ConvLSTM has been widely used in recent years for similar problems [23,24].

In addition to variants of RNN/LSTM, in recent years the Temporal Convolutional Networks (TCNs), an adapted convolutional neural network model for time-series data, are gaining ground for sequence modeling tasks. Typically, Temporal Convolutional Networks refer to a family of 1D-CNN architectures. Their name was first introduced to the literature in the work of [25]. They proposed two types of temporal convolutional models, the Encoder-Decoder TCN and the Dilated TCN, for purposes of fine-grained action segmentation or detection. Their Dilated TCN was an adaptation of the Wavenet model [26]. Wavenet, which was proposed by Google DeepMind, achieved state-of-the-art speech synthesis. It uses a 1D convolutional structure with dilated causal convolutions and includes residual connections, gated activations, skip connections, context stacking and conditioning. In addition, a state-of-the-art performance on character-level language modeling and on English to German translation tasks was also achieved from Google DeepMind ([27]) by using a 1D convolutional network with dilated convolutions and residual blocks, surpassing the performance of recurrent neural networks.

A simple TCN architecture that contains dilated causal convolutions and residual blocks was proposed in [28]. The authors focused on comparing their proposed model to recurrent architectures (LSTM, GRU, and vanilla RNN) in tasks that are commonly used to benchmark RNNs, such as the adding problem, the copy memory task, the JSB Chorales and Nottingham datasets ([29]) and the LAMBADA dataset ([30]) among others. Keeping their TCN and RNN models simple, they conclude that TCNs outperform LSTMs, GRUs and vanilla RNNs. One should note, however, that this is not always the case. For example, in [31], the authors demonstrated that LSTM and CNN achieved better performance compared to TCN day-ahead electricity price forecasting in the Spanish electricity market.

## 3. Materials and Methods

### 3.1. The E-TCN Framework

Formally, the objective in this work is to build a model that receives as input a fixed number of images from a spatial time series and estimates the same number of images shifted by one time step into the future, effectively performing a one-step look ahead prediction. The constant number of the input and output images is a hyperparameter called *timesteps*. Thus, the input data have to be of size timesteps (*N*) × width (*M*) × height (*M*) × number of channels (*c*), while the output data had to be of size timesteps (*N*) × width (*M*) × height (*M*) × number of filters (*f*).

A typical approach when dealing with temporal data, i.e., timeseries, is to employ Long Short Term Memory (LSTM) networks. The inability of typical LSTM networks to take spatial correlations into account was the inspiration for the ConvLSTM model. It was explicitly designed in order to capture both the spatial and temporal dependencies of the dataset. Thus, at the LSTM equations ([32]), matrix multiplications are replaced by convolution operations in the input-to-state and state-to-state transitions. Then, ConvLSTM equations result as below, where ∗ represents the convolution operator and ∘ represents the Hadamard, i.e., element-wise, product:

(1) $$\begin{array}{cc} \; & i_{t}=\sigma \left(W_{xi}*X_{t}+W_{hi}*H_{t-1}+W_{ci}\circ C_{t-1}+b_{i}\right) \end{array}$$

(2) $$\begin{array}{cc} \; & f_{t}=\sigma \left(W_{xf}*X_{t}+W_{hf}*H_{t-1}+W_{cf}\circ C_{t-1}+b_{f}\right) \end{array}$$

(3) $$\begin{array}{cc} \; & C_{t}=f_{t}\circ C_{t-1}+i_{t}\circ tanh\left(W_{xc}*X_{t}+W_{hc}*H_{t-1}+b_{c}\right) \end{array}$$

(4) $$\begin{array}{cc} \; & o_{t}=\sigma \left(W_{xo}*X_{t}+W_{ho}*H_{t-1}+W_{co}\circ C_{t}+b_{o}\right) \end{array}$$

(5) $$\begin{array}{cc} \; & H_{t}=o_{t}\circ tanh\left(C_{t}\right). \end{array}$$

**Figure 1:**
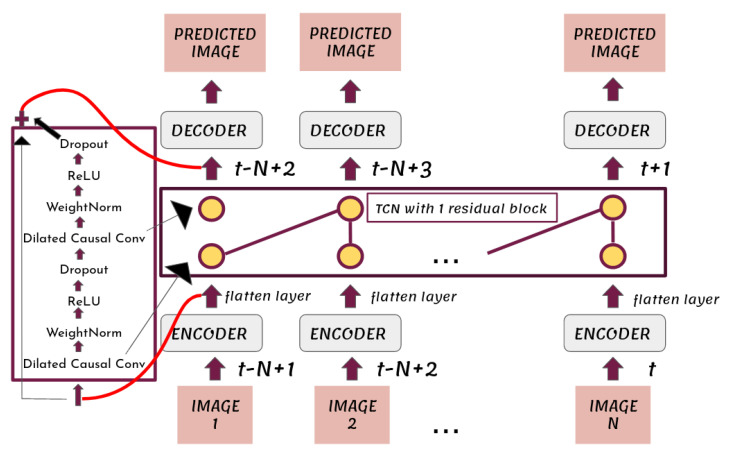
The proposed Embedded Temporal Convolutional Network. The square block at the left shows the inner structure of the used residual block.

In this work, in order to encode information from both spatial and temporal dimensions, we propose the E-TCN, a deep learning model that combines three different networks, an encoder network, a Temporal Convolutional Network inspired from [28] and a decoder network. A high-level visualization of the proposed E-TCN is presented in Figure 1. Formally, the encoder network receives as input a single image, thus its input data is 3-dimensional, M×M×c. To insert multiple images, corresponding to different time-instance, into the model, i.e., to add the dimension of *timesteps* to the input data, the Time Distributed layer of the deep learning framework Tensorflow was wrapped around the encoder network. Figure 1 shows that each of the model’s input images 1,2,…,N at time t−N+1,t−N+2,…,t, respectively, where *N* represents the hyperparameter *timesteps*, passed through an encoder network. To that end, the encoder network consists of three blocks, each block consisting of a 2D convolutional layer followed by a max pooling layer. The role of the max pooling layers is to return the maximum value from a 2×2 pixels image region. The encoder network output *N* 3D vectors. Each of these *N* 3D vectors is passed through a flatten layer which converted them to a set of *N* 1D vectors, preserving the time dimension.

To help in the exposition of the core ideas of the E-TCN, assume that the input is single-channel images of M×M pixels, the dimensions of the 2D convolutional kernels is k×k, and the number of filters in the 2D convolutional layers are A,B and *C*, for the first, second and third block, respectively. The dimensions of the encoder’s input is N×M×M×1. Thus, the dimensions of the output of the first convolutional layer becomes N×M−(k−1)×M−(k−1)×A (for valid padding). The output of the first max pooling layer is thus N×[M−(k−1)]/2×[M−(k−1)]/2×A. If needed, the number of pixels is rounded down to the smallest integer. The output of the second convolutional layer is N×[[M−(k−1)]/2]−(k−1)×[[M−(k−1)]/2]−(k−1)×B and the output of the second max pooling layer is N×[[[M−(k−1)]/2]−(k−1)]/2×[[[M−(k−1)]/2]−(k−1)]/2×B. The output of the third convolutional layer is N×[[[M−(k−1)]/2]−(k−1)]/2−(k−1)×[[[M−(k−1)]/2]−(k−1)]/2−(k−1)×C, which is also the final output of the encoder network.

The encoder network is followed by a Temporal Convolutional Network (TCN) architecture that replaces 1D convolutional layers with residual blocks. The internal structure of the residual block (left block of Figure 1), which was proposed in [28], consists of two 1D convolutional layers of the same kernel size and number of output filters. Each of them is followed by a rectified linear unit activation and a dropout layer. Furthermore, weight normalization ([33]) is applied to the filters of the convolutions. In general, when we use a 1D convolutional layer, its input is a sequence of timesteps vectors. Thus, the *N* 1D vectors which are generated by the flatten layer act as the input of the first residual block of the TCN and subsequently propagated through the different layers. The dimension of each input vector is equal to the number of input channels at the first 1D convolutional layer inside the TCN. The essential point of a residual block is that its input is added invariably to its output. This sum goes through an activation, in our case a rectified linear unit activation, giving the final output of the residual block. The E-TCN that is shown in Figure 1 consists of a single residual block, however, in general may consist of multiple layers.

Unlike typical convolutional networks, the E-TCN uses only causal convolutions, i.e., convolution operations that depend only on past and current information, therefore forcing the prediction at time *t*, $y_{t}$, to depend only on the model’s inputs $x_{o},x_{1},\ldots ,x_{t}$. The predicted image at time *t* therefore only depends on the images at times t−N+1,t−N+1,…,t−1. This is reflected in Figure 1, where the kernel is represented with purple lines between the 2 convolutional layers. As in [28], we also used dilated convolutions. The given input $x_{i}$ of a dilated convolution operation *F* on a component *s* of a layer contains defined gaps of size *d* between its elements. The normal convolution is applied for d=1. The relationship that describes this operation is given by:

(6) $$F\left(s\right)=\sum_{i=0}^{k-1}f\left(i\right)\cdot x_{s-d\cdot i},$$

where *k* is the filter size and f(i) is the *i* element of the applied filter. As it is widely used, the dilation size grew exponentially by 2 at each residual block added to the network. The outputs of the TCN model were *N* 1D vectors at t−N+2,t−N+3,…,t+1. The dimension of each output vector is equal with the number of output filters of the last residual block of the TCN model. We used a reshape layer to turn these 1D vectors to a set of *N* 3D vectors and then a decoder network to turn the 3D vectors to our predicted images.

The decoder network followed the reverse process of the one in the encoder network. It consists of 3 2D transposed convolutional layers between which there were 2 batch normalization layers. The last layer of the decoder network is a 2D convolutional layer with 1 filter, because in this work we only used 1-channel images. The Time Distributed layer is wrapped around the decoder network as before.

We return to our previous example. We suppose that the size of the 2D convolutional kernels at the transposed convolutional layers and the convolutional layer is k×k and the number of filters at the three 2D transposed convolutional layers is C,B and *A*, respectively. In order to predict images of size N×M×M×1 using the above-mentioned structure of the decoder, the output of the reshape layer must be equal to N×M/4×M/4×1. Thus, the output of the TCN is $N\times {\left(M/4\right)}^{2}$, where ${\left(M/4\right)}^{2}$ is the number of output filters of the last residual block of the TCN model and is set by us. The output of the first transposed convolutional layer is N×M/2×M/2×C. The output of the second transposed convolutional layer is N×M×M×B and the output of the third transposed convolutional layer is N×M×M×A. The output of the final 2D convolutional layer is of size N×M×M×1.

### 3.2. Analysis Ready Dataset

We quantify the performance of the proposed scheme by measuring the accuracy in the estimation of essential climate variables. Specifically, the proposed (E-TCN) and the state-of-the-art method (ConvLSTM) are trained using time series of historical observations and learn to predict future values of Land Surface Temperature and Surface Soil Moisture, derived by compositing and averaging the daily values from the corresponding month. The datasets considered in this work were created from single channel satellite derived products obtained from the NASA worldview application (https://worldview.earthdata.nasa.gov/, accessed on 1 June 2021).

The land surface temperature values correspond to level 3 (L3) products extracted from the MODIS Terra satellite. Specifically, the Land Surface Temperature/Emissivity layer encodes monthly averages of daytime land surface temperature in Kelvin (K) (Land Surface Temperature layer, https://worldview.earthdata.nasa.gov/?v=-152.8838071329185,-16.82356580605071,50.20782633321695,82.73893419394929&l=MODIS_Terra_L3_Land_Surface_Temp_Monthly_Day&lg=true&t=2021-09-22-T18, accessed on 1 June 2021). These estimations are provided by the day/night algorithm which considers several TIR bands [34]. For the soil moisture, we consider the NLDAS estimated soul moisture derived by the North America Land Data Assimilation System Phase 2 Mosaic Land Surface Model. The NLDAS Soil Moisture monthly data are generated through temporal averaging of the hourly data and represents the 0–10 cm depth-averaged amount of water.

**Figure 2:**
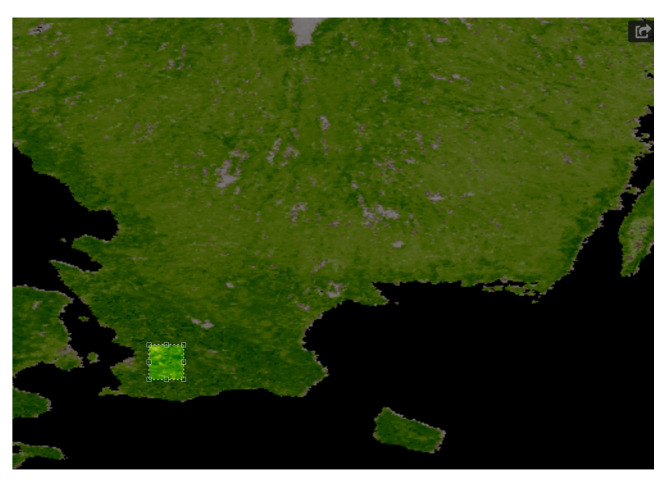
The square shows the area in Sweden from which we obtained land surface temperature in images of 28×28 pixels, with a resolution (per pixel) of 1 km.

**Figure 3:**
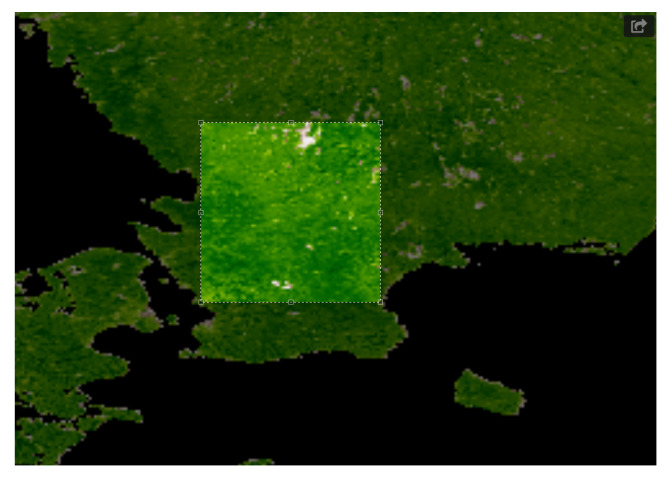
The square shows the area in Sweden from which we land surface temperature in images of 28×28 pixels, with a resolution (per pixel) of 5 km.

**Figure 4:**
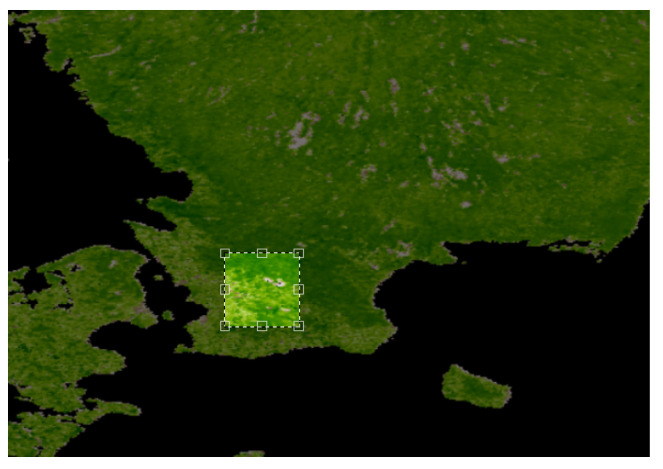
The square shows the area in Sweden from which we obtained land surface temperature in images of 60×60 pixels, with resolution (per pixel) of 1 km.

**Figure 5:**
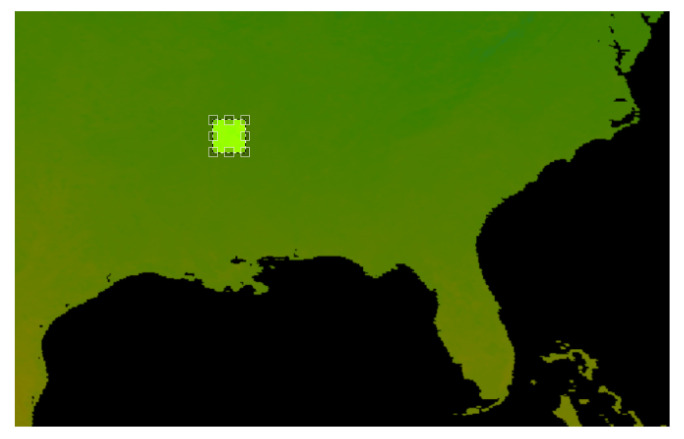
The square shows the area in USA from which we obtained our images of 140×140 pixels, with resolution (per pixel) of 1 km.

For each experiment, we split the full dataset into two separate sets, the training and the test dataset. These dataset consists of images representing values of daytime land surface temperature from January 2003 to May 2020 and for soil moisture from April 2015 to May 2020, respectively. For both cases, the objective is the prediction of the corresponding values for June of 2020. We compiled four datasets to test the ability of the models to predict land surface temperature values, each consisted of 210 examples, and more specifically:

- A set of 28×28 pixel images with per pixel resolution equal to 5 km. These images were acquired from the region in Idaho shown in Figure 2.
- A set of 28×28 pixel images with per pixel resolution equal to 5 km. These images were acquired from the region in Sweden shown in Figure 3.
- A set of 60×60 pixel images with per pixel resolution equal to 1 km. These images were acquired from the region in Sweden shown in Figure 4.
- A set of 140×140 pixel images with per pixel resolution equal to 1 km. These images were acquired from the region in USA shown in Figure 5.

**Figure 6:**
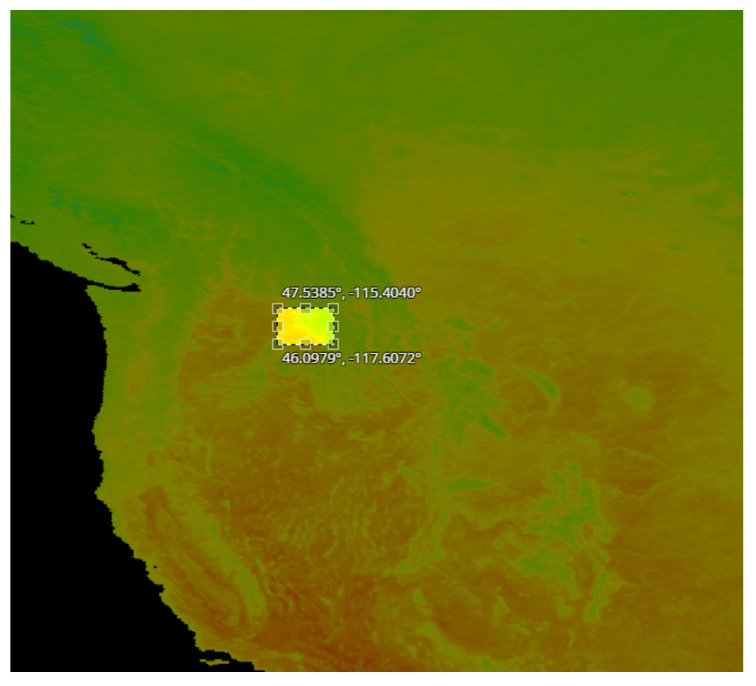

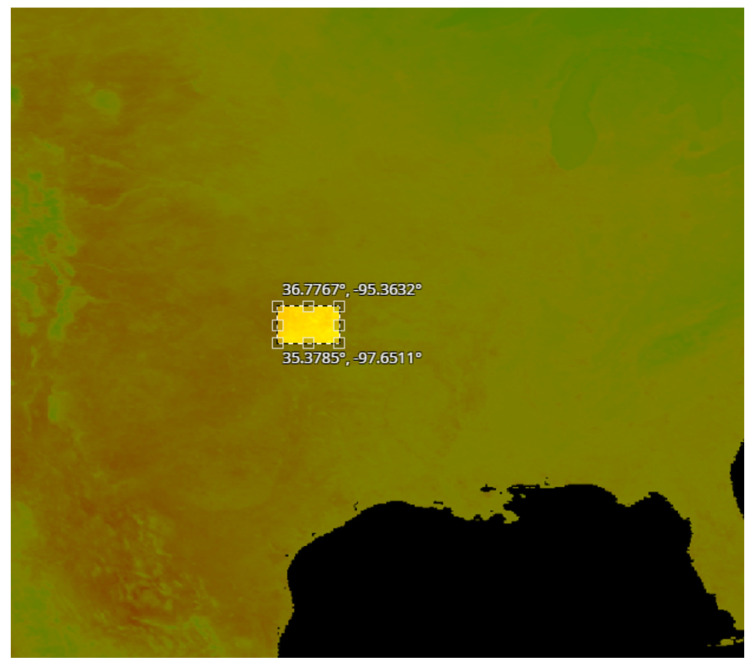
The squares shows the area in Idaho (**top**) and Arkansas (**bottom**) from which we obtained soil moisture values, with resolution of 5 km per pixel.

Additionally, we used two datasets to test the ability of the two models to predict values of the soil moisture. Each of them consisted of 15 a set of 28×28 pixel images with per pixel resolution equal to 5 km encoding surface soil moisture. These images were acquired from two regions in USA, Idaho and Arkansas shown in Figure 6.

## 4. Results

### 4.1. Performance Evaluation Metrics

To quantify prediction accuracy, we employ a number of metrics, including the correlation and reconstruction error. Formally, let $y_{i}$ and $x_{i}$ represent the pixels of the predicted and the real values, respectively, $\bar{x}$ and $\bar{y}$ are the mean values and *n* represents the total number of pixels. The Pearson correlation coefficient (PCC) is used for comparative purposes, which is given by the equation:

(7) $$\mathrm{PCC}=\frac{\sum_{i=1}^{n}\left(x_{i}-\bar{x}\right)\left(y_{i}-\bar{y}\right)}{\sqrt{\sum_{i=1}^{n}{\left(x_{i}-\bar{x}\right)}^{2}}\sqrt{\sum_{i=1}^{n}{\left(y_{i}-\bar{y}\right)}^{2}}}.$$

The PCC takes values between −1 and 1 and indicates the extent to which two datasets are linearly related. PCC = 1 indicated that the pixels between the predicted and the ground truth images are total positive linear correlated, while if PCC = −1 the pixels between these two images are total negative linear correlated. In addition to PCC, three more indexes encoding pixel-level errors were selected to measure the performance of the two different models, the mean square error (MSE), the mean absolute error (MAE) and the unbiased root mean squared error (ubRMSE), which are defined as follows:

(8) $$\mathrm{MSE}=\frac{1}{n}\sum_{i=1}^{n}{\left(x_{i}-y_{i}\right)}^{2},$$

(9) $$\mathrm{MAE}=\frac{1}{n}\sum_{i=1}^{n}\left|\left(x_{i}-y_{i}\right)\right|,$$

and

(10) $$\mathrm{ubRMSE}=\sqrt{\left(\frac{1}{n}\sum_{i=1}^{n}{\left(x_{i}-y_{i}\right)}^{2}-\frac{1}{n}\sum_{i=1}^{n}{\left(\frac{1}{n}\sum_{i=1}^{n}x_{i}-\frac{1}{n}\sum_{i=1}^{n}y_{i}\right)}^{2}\right)}.$$

For the three cases of pixel-level errors, values closer to zero indicated better performance.

### 4.2. Ablation Study

In order to understand the influence of the different design choices, an ablation study is performed by changing the hyperparameters that structured the E-TCN. These are the *timesteps*, the number of filters in each of the three 2D convolutional layers at the encoder part, the kernel size of the 1D and 2D convolutional layers, the dropout rate, the number of the residual blocks and the number of their filters. We present the results of the ablation study on the daytime land surface temperature (Figure 2) which was acquired from a region in Sweden and consisted of 28×28 pixels satellite images at 1km resolution.

**Figure 7:**
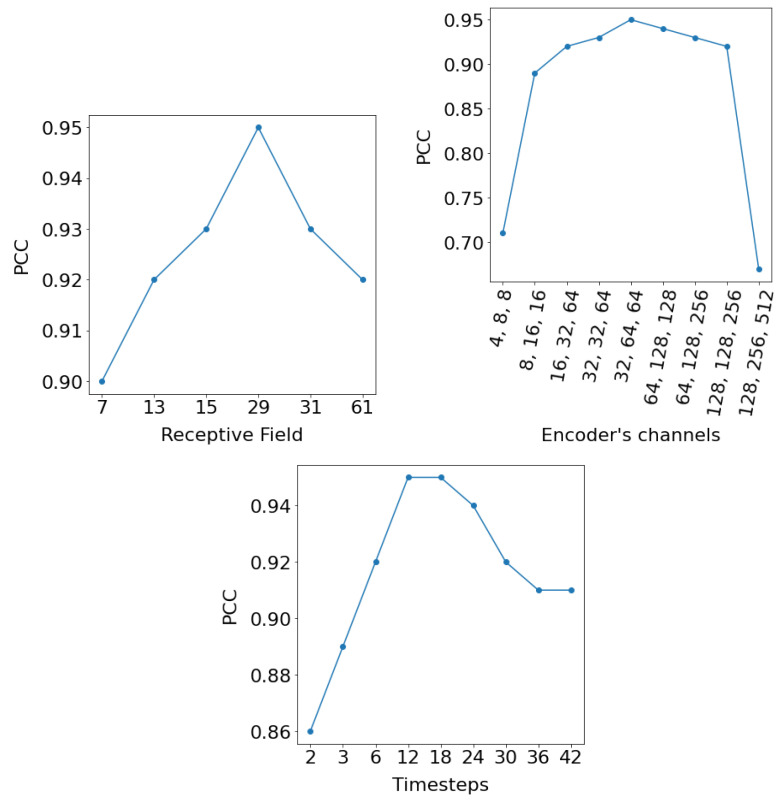
PCC as a function of the receptive field (**top left**), the number of filters in each of the 2D convolutional layers at the encoder part (**top right**), and the hyperparameter *timesteps* (**bottom**).

Figure 7(top left) presents the PCC as a function of the receptive field. The receptive field of a TCN with *n* residual blocks, fixed kernel size, *k*, and exponentially increasing dilations, is given by:

(11) $$1+2\times \left(k-1\right)\times \left(2^{n}-1\right)$$

at the final block. Thus, in order to test the dependence of the PCC from the receptive field, we were changing the kernel size and the number of the residual blocks. Results indicate that the relationship between receptive field size and performance is relatively stable (above 0.9), however, it is not monotonic but characterized by specific “optimal” configurations. Figure 7(top right) also reports the PCC as a function of the number of filters in each of the three 2D convolutional layers at the encoder part. Results in this case indicate that “extreme” configurations, either very small or very large architectures suffer a dramatic loss in performance. Last, in Figure 7(bottom) we investigate the impact of the timesteps hyperparameter which relates to the amount of historical information needed for forecasting. Results indicate that, at first, increasing the number of previous observations leads to higher performance, while from some onward, performance is reduced, presumably because the networks start to overfit to specific conditions.

A similar process was followed for each experiment and the final model was chosen as the one that consisted of the hyperparameters which resulted the highest PCC between the ground truth and the predicted values. The set of hyperparameters employed for each scenario are encoded in Table 1.

### 4.3. Prediction of Land Surface Temperature at Various Resolutions

In this subsection, we employ the proposed E-TCN and the state-of-the-art ConvLSTM for the prediction of the daytime land surface temperature in June 2020 and explore two scenarios by changing the spatial resolution. In the first case, the spatial resolution is equal to 1 km (Figure 2), while for the second it is 5 km (Figure 3). The objective is to explore the model’s response over different size areas while keeping the number of pixels (unknowns) constant. We extracted data from the same, although enlarged, area in Sweden. Thus, the climate characteristics in the two experiments are expected to be similar. In this way, we were able to assert if the performance of the deep learning models is dependent on the spatial resolution.

In Section 4.2, we asserted that the best model for the dataset with spatial resolution 1km is shown in Table 1 (Case 1). Repeating the same process, the hyperparameters that were used in the E-TCN for the dataset with spatial resolution 5 km are shown in Table 1 (Case 2).

For the case of the ConvLSTM, in each of the two layers, 64 filters were used. The ConvLSTM model which was used on the dataset with spatial resolution equal to 5 km consisted of one ConvLSTM layer with 100 filters and a 3D convolutional layer as the output layer. In both models, the hyperparameter timesteps was equal to 18 and the size of the convolutional kernels at the ConvLSTM layers was 3×3 while at the 3D convolutional layer is 3×3×3.

The PCC, the MSE, the MAE, as well as the ubRMSE between the predicted and the true values of the daytime land surface temperature in June 2020 as they resulted when each of the two models was used, are presented in Table 2. Comparing the two cases of one and five km region, results indicate that both methods suffer from a performance drop where larger areas are considered. In both cases however, the proposed E-TCN model is able to achieve higher performance across all metrics. This table also includes the number of the trainable parameters of the selected models, as well as the training time in msec per epoch. These results indicate that the requirements of the ConvLSTM model are more demanding compared to the E-TCN which achieves higher PCC with fewer parameters.

**Figure 8:**
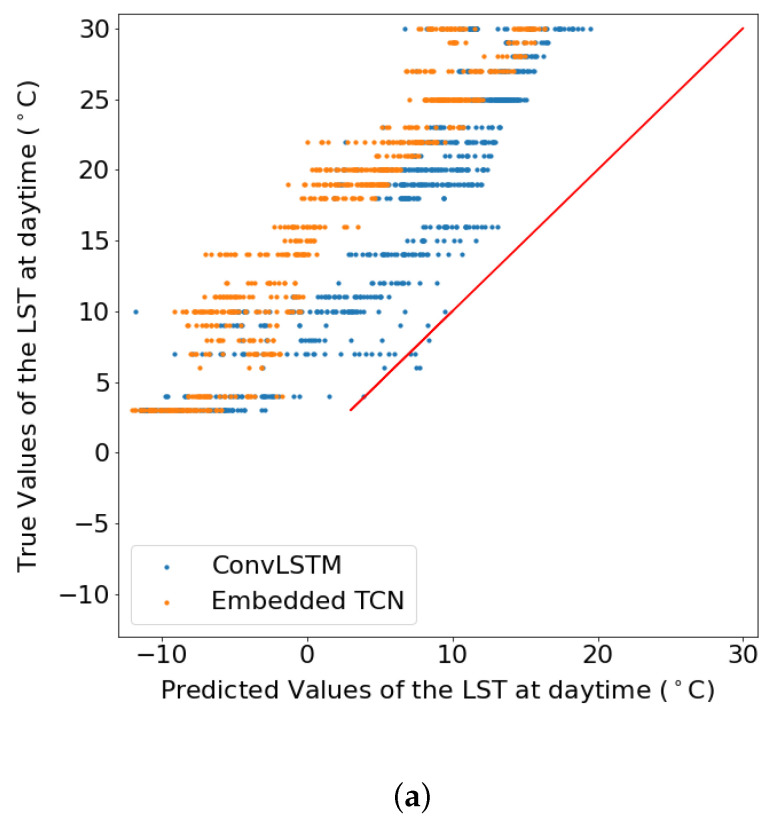

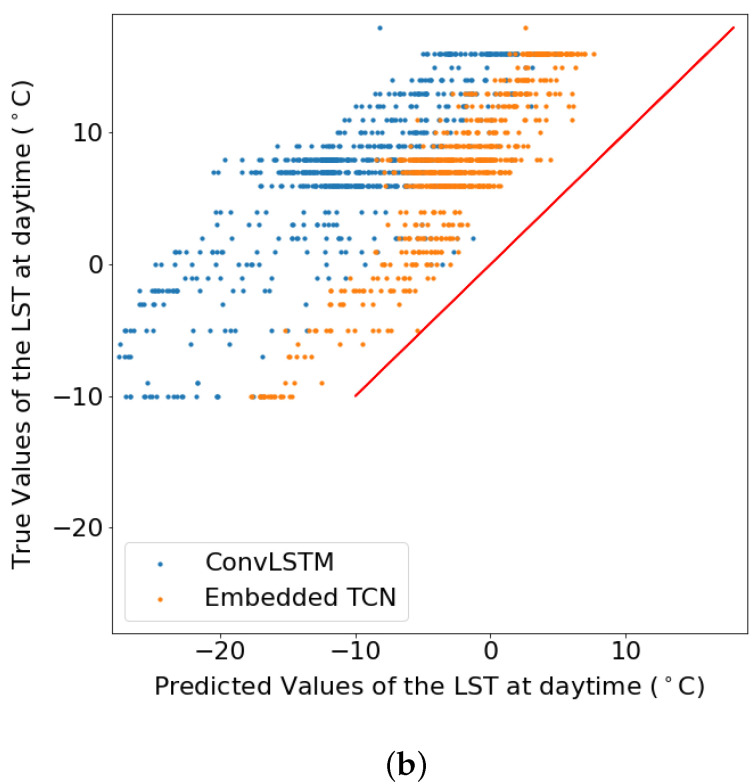
The true values of the daytime land surface temperature in June 2020 as a function of the predicted values when the dataset consisted of 210 images of 28×28 obtained from a region in Sweden with resolution (per pixel) (**a**) 1 km, (**b**) 5 km. (**a**) LST prediction for 1 km spatial resolution. (**b**) LST prediction for 5 km spatial resolution.

In addition to Table 2, Figure 8 illustrates the scatter plots between the true and the predicted values which emerged from each model for the two scenarios. This results suggest that especially for the case of 1km spatial resolution, both methods perform poorly, but in this case, ConvLSTM performs marginally better. For the case of 5km, the proposed E-TCN predicted values are much closer to the diagonal which indicates the ideal performance, compared to the ConvLSTM.

### 4.4. Dependence on the Number of Training Examples

In addition to performance as a function of spatial resolution, we also test the dependence on the number of training data by changing the number of training examples. First, we set them equal to 89 and then equal to 149. As before, we were aiming the prediction of the daytime land surface temperature in June of 2020. Table 3 shows the resulting PCC according to the size of the training dataset for both models. The Pearson correlation coefficient did not change significantly as the number of training images increased when the E-TCN is used. In contrast, the PCC increased more distinctly as a function of the number of training images, when the ConvLSTM model is used. The significantly higher sensitivity of the ConvLSTM model can be attributed to the larger number of parameters employed in the model, compared to the more robust E-TCN.

### 4.5. Impact of Region Size

In order to study the impact of the region size, i.e. input patch size, two experiments were performed where the size of the images were 60×60 pixels and 140×140 pixels, respectively. In the first experiment, the dataset consisted of 210 images depicting the daytime land surface temperature in an area of Sweden (Figure 4). Their resolution (per pixel) was equal to 1 km. The hyperparameters selected for our model are shown in Table 1 (Case 3). In addition, we used a ConvLSTM model. It consisted of two layers of 100 filters, the timesteps was set to 8, the kernel’s size at the ConvLSTM layers was set to 3×3 while at the 3D convolutional layer was set to 3×3×3. Table 4 summarizes the results of the comparison between the two models with respect to performance, number of parameters and training time. Comparing the two methods, we can once more observe that the proposed scheme achieves higher prediction quality while having lower processing requirements. We also note that in comparison with smaller patches, i.e., comparing with the performance reported in the top two rows in Table 2, performance degrades with larger patch sizes.

We also studied a larger region of 140×140 pixels in south-east USA (Figure 5). The dataset consisted of 210 satellite images with resolution (per pixel) equal to 1 km. We split the area of 140×140 pixels in 25 subareas of 28×28 pixels. Thus, we created 25 datasets of 210 images. The goal was the prediction of 25 images of 28×28 pixels depicting the daytime land surface temperature in June 2020. The E-TCN as well as the ConvLSTM model were used to predict each of these images, using the same hyperparameters for all of the 25 times the networks were trained. The hyperparameters of the E-TCN are presented in Table 1 (Case 5). The ConvLSTM model consisted of two ConvLSTM layers of 64 filters, followed by a 3D convolutional layer while the hyperparameter timesteps was equal to 18. The size of the convolutional kernels at the ConvLSTM layers was 3×3 and at the 3D convolutional layer was 3×3×3. As before, we computed the PCC, the MSE, the MAE, and the ubRMSE between the predicted values and the ground truth. Table 5 presents the average of these metrics over the 25 different subareas.

**Figure 9:**
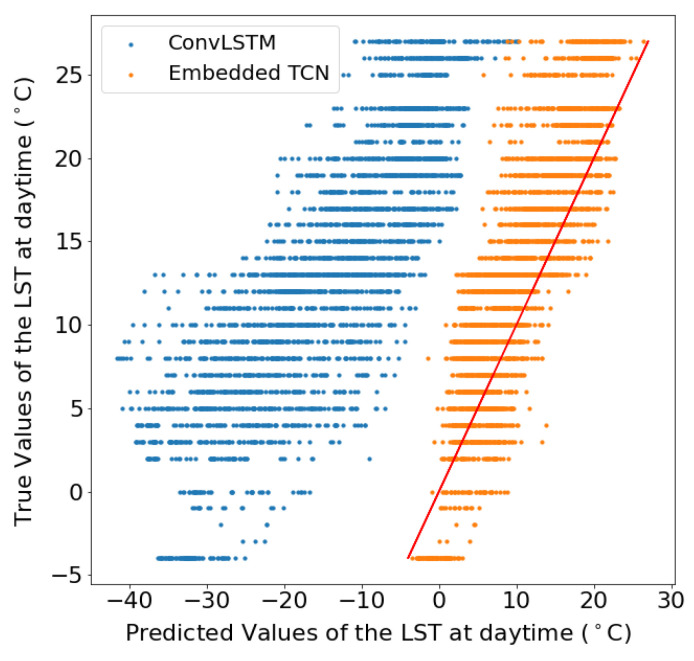
LST scatter plot for 60×60 pixels images.

**Figure 10:**
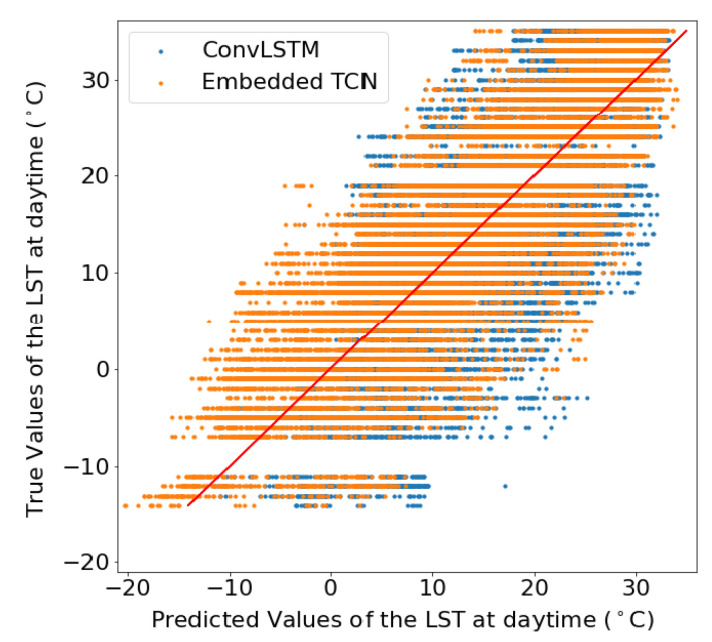
LST scatter plot for 140×140 pixels.

Figure 9 and Figure 10 present the scatter plot between the true and the predicted values from two different spatial sizes. The figures show the true values of the land surface temperature (LST) at daytime in June 2020 as a function of the predicted values for datasets of 60×60 pixels from a region in Sweden and 140×140 pixels for a region in Sweden, respectively.

These results showcase the superiority of the proposed E-TCN compared to the ConvLSTM for the case of daytime LST estimation. The performance gains are substantially better for the region in Sweden where we observe a very high correlation between predicted and estimated LST, while the competing ConvLSTM completely fails to capture the dynamics.

### 4.6. Evaluation on Different Time Instances

**Figure 11:**
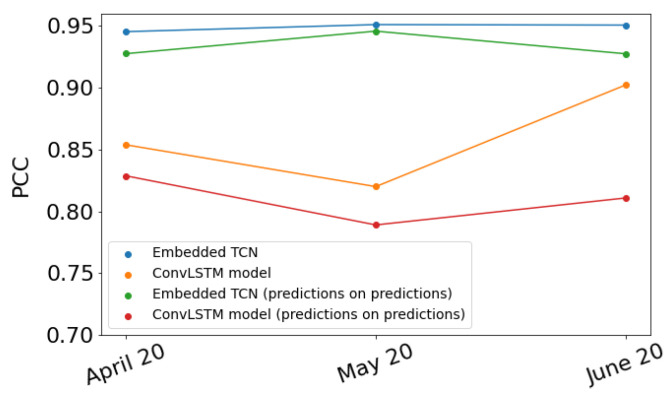
PCC between the predicted values and the ground truth in April, May and June of 2020.

Up to this point, this work focused on the prediction of values of the daytime land surface temperature in June 2020. We repeated the process described above to predict the values of the daytime land surface temperature in April 2020 and May 2020. In each of these cases, we reduced the number of training images by two and one, respectively. In Figure 11, the blue dots show the PCC between the predicted and the true values for each of the three months when the E-TCN was used while the orange dots show the PCC when the ConvLSTM model was used.

**Figure 12:**
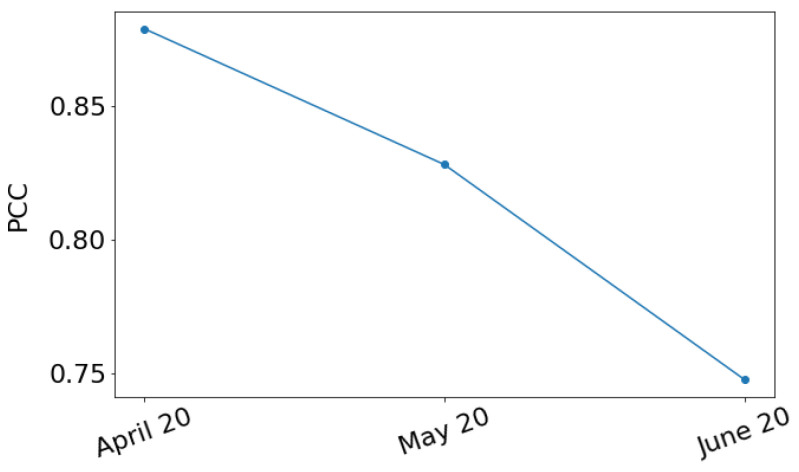
PCC for different the *timesteps* predicted images and the *timesteps* true images as a function of the last month of the *timesteps* predicted images.

Recall that to predict the values in March 2020, both of our models used *timesteps* images and predicted *timesteps* images shifted by one time step. The last predicted image represents the values of the daytime land surface temperature in March 2020. Thus, finally, we used these *timesteps* predicted images as input to our models in order to predict *timesteps* images where the latter represents the values of the daytime land surface temperature in April 2020. We repeated this process three times in total. The green and red dots in Figure 11 show the PCC resulted following this process when the E-TCN and the ConvLSTM model is used, respectively, for each of the three months. This process is usually referred to as “predictions on predictions” method. For each month, the PCC is smaller when we used the “predictions on predictions” method than when we used the method described in the above paragraphs. Figure 12 presents the PCC between the *timesteps* predicted images and the *timesteps* true images when the E-TCN is used.

In addition to the previous scenarios where performance is quantified for the prediction of a single month, we also explore the application of the proposed and the state-of-the-art method in using all available monthly measurements between 2003 and 2019 for training and predicting each month of 2020 independently.

**Figure 13:**
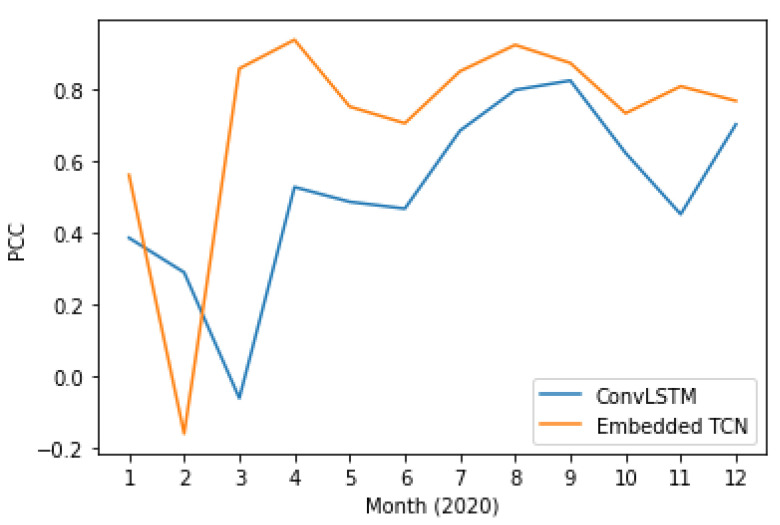
Pearson correlation coefficient between proposed and the state-of-the-art method for LST prediction over different months of 2020.

**Figure 14:**
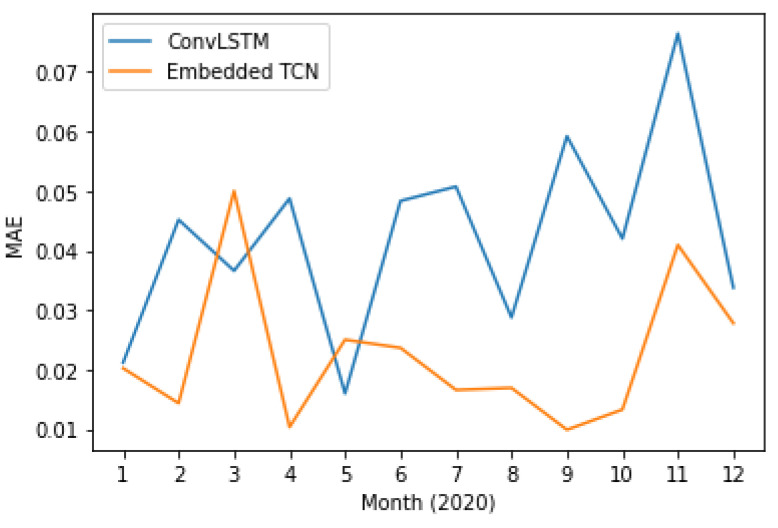
Mean absolute error between proposed and the state-of-the-art method for LST prediction over different months of 2020.

**Figure 15:**
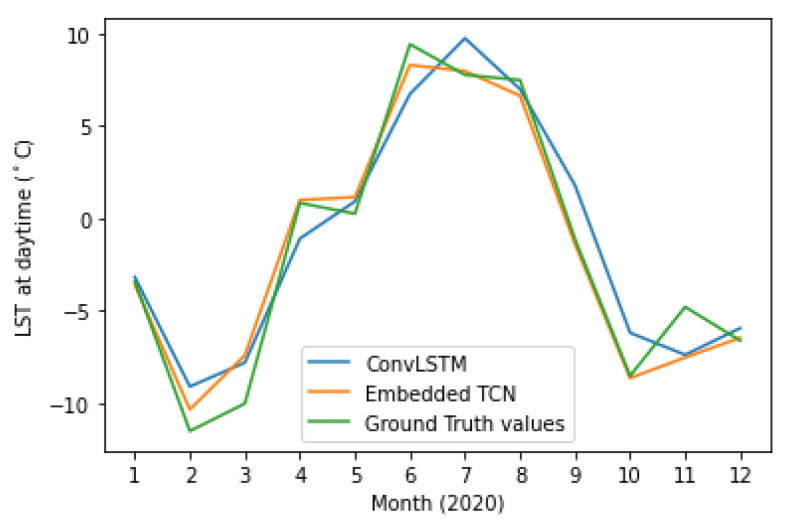
Timeseries of actual and predicted LST over different months of 2020.

Figure 13 and Figure 14 present the PCC and MAE between the predicted and the actual LST values. This case also supports the evidence of significantly better performance of the proposed E-TCN scheme compared to the ConvLSTM approach. In order to appreciate the merits of each method, Figure 15 presents the ground-truth, the proposed E-TCN and state-of-the-art ConvLSTM. Overall, we observe that both methods are able to capture phenomena like seasonality, while the proposed method’s prediction are closer to the actual ground-truth.

### 4.7. Prediction of Soil Moisture

**Figure 16:**
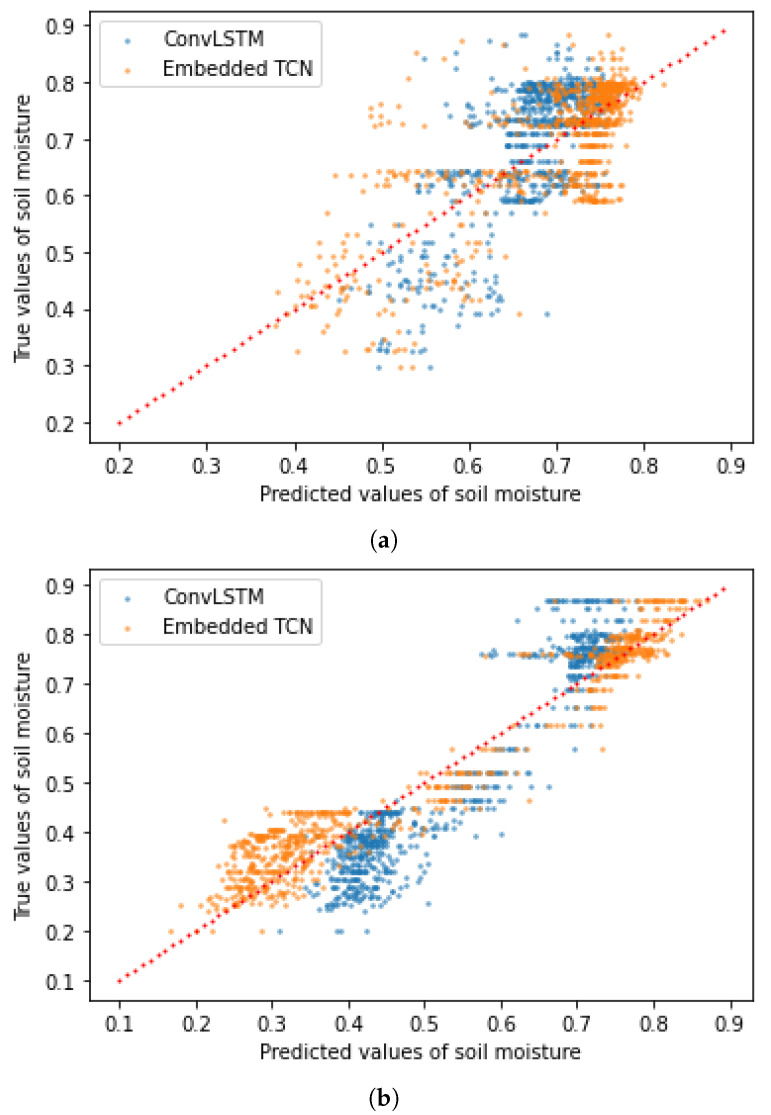
The true values of the soil moisture in June 2020 as a function of the predicted values when the dataset consisted of images of 28×28 at 1 km per pixel resolution, obtained from a region in (**a**) Idaho and (**b**) Oklahoma. (**a**) Predictions scatter plot for a region in Ihado. (**b**) Predictions scatter plot for a region in Oklahoma.

In the experiments we also considered predicting values of the soil moisture using a datasets of 150 images of 28×28 pixels with spatial resolution of 6 km. Our goal was the prediction of the values of the soil moisture in June 2020. We repeated the experiment in two different regions in the USA, in one region in Idaho and in one region in Arkansas in order to capture a large diversity of climate pattern. We consider the proposed E-TCN and the state-of-the-art ConvLSTM model for the prediction of soil moisture values. The hyperparameters of the E-TCN are presented in Table 1 (Case 4). The ConvLSTM model which was applied on data from Sweden consisted of two layers of 60 filters and the timesteps was set to 18 while the one which was applied on data from Greece consisted of one layer of 84 filters and the timesteps was set to eight. In both cases, the kernel’s size at the ConvLSTM layers was set to 3×3 while at the 3D convolutional layer, it was set to 3×3×3. Table 6 summarizes the results of the comparison between the two models in both regions while Figure 16 shows the scatter plots between the true and the predicted values.

We observe that the PCC is higher when the E-TCN is used than when the ConvLSTM model is used, while the mean square error and the mean absolute error were smaller, regardless of the area from which we obtained our data. However, the three metrics between the predicted values and the ground truth in the area of Sweden are better than in the region of Greece. This fact is an indication that the area from which we obtain our data is important for the performance of the two models.

## 5. Discussion

In the previous section, we performed a thorough investigation of the different aspects of the proposed scheme and compared the performance to a state-of-the-art method for forecasting soil moisture and land surface temperature. Overall, experimental results indicate that both data-driven approaches, namely ConvLSTM and the proposed E-TCN, are able to predict the values. For both methods, we observed the following behaviour:

- Higher spatial resolution makes the problem more challenging.
- Prediction of large areas leads to better prediction accuracy.

With respect to the performance of the proposed E-TCN, compared to ConvLSTM, we observed that:

- E-TCN achieved higher overall performance for both soil moisture and surface temperature.
- E-TCN achieved better performance in term of prediction quality with respect to training set size.
- E-TCN are characterized by a smaller number of parameters, and thus less prone to overfitting.
- E-TCN is a more compact network (in term of network parameters), making it more appealing for real-time/large-scale applications.

Lastly, to corroborate the argument that the proposed scheme achieves better performance compared to the state-of-the-art, we also considered a statistical test based on a dependent t-test. For the case of LST at 1 km over Sweden, the *p*-value is *p* = 3 × 10${}^{-19}$, indicating that the null hypothesis, i.e., that the two methods performance (on average) the same, is not true, while similar results also hold for the other cases.

## 6. Conclusions

Forecasting of values of ECV given currently available satellite observations can be critical for predicting the evolution of climate and adopting appropriate strategies. In this work, we consider the paradigm shifting framework of data-driven prediction, and explore how deep learning models can support the accurate estimation of critical variables like surface temperature and soil moisture. Our model essentially offers the high-dimensional extension of the temporal convolutional neural network architecture through the introduction of an encoder and a decoder. To quantify the performance, we consider a wide range of locations and different products. Experimental results demonstrate that the proposed scheme outperforms the state-of-the-art time-series prediction architectures (ConvLSTM) in terms of prediction accuracy, while also requiring fewer parameters. Future work will expand on the number of ECVs and demonstrate the merits of data-driven ECV forecasting approaches in different problems like olive phenology phase prediction [35].

## Figures and Tables

**Table 1:** Table to show the hyperparameters that were chosen for the E-TCN for each of the experiments. W represents the hyperparameter *timesteps* and Drop., the dropout rate. The third column shows the number of filters at the three 2D convolutional layers in the encoder network.

Case	W	Encoder’s Filters	2D Conv Kernel	1D Conv Kernel	TCN	Drop
1	12	32, 64 & 64	(4, 4)	3	3 residual blocks of 64, 49 & 49 filters	0.3
2	8	48, 48 & 96	(4, 4)	2	3 residual blocks of 96, 49 & 49 filters	0.3
3	12	8, 16 & 16	(6, 6)	2	3 residual blocks of 225, 225 & 225 filters	0.3
4	10	32, 64 & 64	(4, 4)	4	3 residual blocks of 64, 49 & 49 filters	0.3
5	18	32, 64 & 64	(4, 4)	4	3 residual blocks of 64, 64 & 49 filters	0.4

**Table 2:** Error metrics for the predicted **land surface temperature** values, number of trainable parameters and training time (in msec per step), when the dataset consisted of images of 28×28 pixels obtained from a region in Sweden. The second column shows the spatial resolution (S.R.) of the dataset.

Model	S.R.	PCC	MSE	ubRMSE	Parameters	Training Time
E-TCN	1 km	0.95	0.00091	0.0053	258,659	50–70
ConvLSTM	1 km	0.90	0.00052	0.0072	446,913	430–450
E-TCN	5 km	0.89	0.00038	0.0053	291,136	40–60
ConvLSTM	5 km	0.82	0.00120	0.0077	366,701	340–360

**Table 3:** PCC for different number of training images as resulted by using the E-TCN and the ConvLSTM model.

Number of Training Images	PCC (E-TCN)	PCC (ConvLSTM Model)
89	0.94	0.85
149	0.93	0.87
209	0.95	0.90

**Table 4:** Error metrics, number of trainable parameters and training time (msec per step) for the dataset consisted of images of 60×60 pixels with 1 km per pixel obtained from a region in Sweden.

Model	PCC	MSE	ubRMSE	Parameters	Training Time
E-TCN	0.85	0.000092	0.0078	926,920	100–130
ConvLSTM	0.83	0.0032	0.012	1,087,101	2000

**Table 5:** The average PCC, MSE and ubRMSE between the predicted values and the ground truth over the 25 predicted images. This table also illustrates the number of trainable parameters and the training time in msec per step both for the E-TCN and the ConvLSTM mode when the dataset consisted of images of 140×140 pixels.

Model	PCC	MSE	unRMSE	Parameters	Training Time
E-TCN	0.78	0.0018	0.028	277,190	50–60
ConvLSTM	0.59	0.0026	0.039	446,913	350–390

**Table 6:** Error metrics for the predicted **soil moisture** values and number of trainable parameters.

Model	PCC	MSE	ubRMSE	Parameters
E-TCN (Idaho)	0.74	0.0062	0.0828	343,814
ConvLSTM (Idaho)	0.71	0.0076	0.0669	446,913
E-TCN (Arkansas)	0.97	0.0431	0.0030	343,814
ConvLSTM (Arkansas)	0.95	0.0738	0.0078	446,913

## Data Availability

The data presented in this study are openly available in Tsagkatakis, Grigorios (2022): E-TCN dataset. figshare. Dataset. at https://doi.org/10.6084/m9.figshare.19236876.v2 accessed on 1 December 2021, reference number 10.6084/m9.figshare.19236876.v2.
